# Twelve new metagenome-assembled genomes from non-axenic culture of *Griffithsia monilis* (Rhodophyta)

**DOI:** 10.1128/mra.00728-23

**Published:** 2023-12-01

**Authors:** Yanã C. Rizzieri, August Lipari, Laura Gunn, Fay-Wei Li

**Affiliations:** 1 Boyce Thompson Institute, Ithaca, New York, USA; 2 Plant Biology Section, Cornell University, Ithaca, New York, USA; 3 Grinnell College, Grinnell, Iowa, USA; 4 Department of Cell and Molecular Biology, Uppsala University, Uppsala, Sweden; Montana State University, Bozeman, Montana, USA

**Keywords:** metagenomic

## Abstract

We report 12 metagenome-assembled genomes from a non-axenic culture of the red alga *Griffithsia monilis* Harvey, some of which are distantly related to publicly available genomes.

## ANNOUNCEMENT

Nature’s carbon-fixing enzyme, ribulose-1,5-bisphosphate carboxylase/oxygenase (Rubisco), exhibits a slow catalytic turnover rate and poor substrate specificity. One notable outlier is the Rubisco found in the marine red alga *Griffithsia monilis* (1). Transplanting *G. monilis* Rubisco into a C3 chloroplast could boost plant growth by as much as 30% (2) and has been an area of active research (3 – 5). We set out to generate a reference genome for *G. monilis*, but analyses of the preliminary data indicated that our culture was not axenic. Here, we report the metagenomic composition of the culture, from which 12 highly complete bacterial genomes were assembled.

Our *G. monilis* cultures, from Sydney Harbor (Australia), were grown at room temperature in liquid Provasoli enriched seawater medium under indirect light illumination. For DNA extraction, we ground the tissue in liquid nitrogen using mortar and pestle, followed by a modified cetyltrimethylammonium bromide (CTAB) method as described in reference (6 ). Illumina library preparation and pair-end sequencing (150 bp) were done by NovoGene Corp, using their in-house protocol. A total of 33 Gb were generated using NovaSeq6000. For nanopore sequencing, DNA libraries were prepared using the Ligation Sequencing Kit (SQK-LSK109) and sequenced on MinION R9 flowcells (FLO-MIN106D). Basecalling using guppy (v6.0.0) (7) on a SUP mode resulted in a total of 7.6 Gb of nanopore data with a read length N50 of 7,463 bp.

For all programs used, default parameters were used except where otherwise noted. The raw Illumina and nanopore reads were trimmed using fastp (v.0.23.2) (8) and Porechop (v.0.2.4) (9), respectively. To assemble the nanopore reads, we used Flye (v.2.9.2) (10) with the --metagenome flag. The resulting contigs were filtered using Seqkit (v2.4.0) (11) and polished with the Illumina reads using a combination of Medaka (v.1.8.0) (12), Polca (v.4.1.0) (13), and Polypolish (v.0.5.0) (14). Classification of the contigs using Kraken2 (15) suggested the majority of them were of bacterial origin and only 4.3% belonged to Rhodophyta. To characterize the bacteria present in the sample, we used Metabat2 (v.2.12.1) (16) to create metagenomic bins, CheckM (v.1.2.1) (17) to assess the metagenome-assembled genomes (MAGs) completeness, and GTDB-TK (v.2.1.1) (18) to taxonomically classify them. Lastly, we used Bowtie2 (v.2.2.5) ( 19 ) to calculate coverage, and NCBI PGAP (v.1.2) for genome annotation (20). To visualize circular genomes, we used Bandage (v.0.9.0) (21).

A total of 58 bacterial MAGs were recovered, 12 having a completeness score above 95% and less than 4.3% contamination (Table 1). Length varied from 2.4 to 9.4 Mb, with protein-coding genes ranging between 2,421 and 7,493. Nine of the twelve MAGs were recovered as a single contig, whereas eight were circular. Only two MAGs (Padam38 and Padam52) had ANI above 95% when compared to the GTDB-TK database, while others were all divergent including six having ANI lower than 80%.

**TABLE 1:** Sequencing and assembly information for the twelve metagenome-assembled genomes from *Griffithsia monilis* culture

Raw files and general assembly statistics					MAG statistics											
File name	No. of reads	Total size (b)	No. of contigs	N50 (b)	Accession no.	Genome name	Circular genome	fastANI GTDB-TK	Classification from GTDB-TK	Total length (b)	GC content (%)	No. of contigs	Completeness (%)	Contamination (%)	Coverage (x)	No. of protein-coding genes
assembly_polished1k_.fasta	–^*a*^	278,655,119	4,485	155,176	JAWPML000000000	Padam16	Yes	88.22	Bacteria, Actinomycetota, Acidimicrobiia, Acidimicrobiales, UBA11606, JAJRXC01	4,481,363	69.38	1	98.29	1.28	16	4,307
raw_nanopore	1,619,944	7,623,654,891	–	7,463	JAWPMM000000000	Padam2	Yes	80.66	Bacteria, Bacteroidota, Bacteroidia, Cytophagales, Cyclobacteriaceae, Reichenbachiella	4,878,078	40.06	1	98.59	1.79	60	4,134
Illumina_paired	219,587,052	32,913,609,279	–	–	JAWPMN000000000	Padam27	No	83.81	Bacteria, Pseudomonadota, Alphaproteobacteria, UBA8366GCA-2696645, Pacificispira	4,637,961	62.29	1	100	0.43	33	4,321
					JAWPMO000000000	Padam3	No	79.03	Bacteria, Actinomycetota, Acidimicrobiia, Acidimicrobiales, Ilumatobacteraceae, Ilumatobacter	4,968,626	68.28	1	99.15	4.27	39	4,613
					JAWPMP000000000	Padam36	Yes	89.05	Bacteria, Pseudomonadota, Alphaproteobacteria, Parvibaculales, CGMCC-115125, Pyruvatibacter	3,490,971	58.34	1	99.78	0	58	3,276
					JAWPMQ000000000	Padam38	No	96.2	Bacteria, Pseudomonadota, Alphaproteobacteria, Rhodobacterales, Rhodobacteraceae, Roseobacter, Roseobacter sp015644665	4,376,676	60.63	13	99.43	0.06	17	4,203
					JAWPMR000000000	Padam4	Yes	79.85	Bacteria, Pseudomonadota, Alphaproteobacteria, Rhodobacterales, Paracoccaceae, Roseobacter	5,082,563	55.4	1	98.78	0.43	37	4,561
					JAWPMS000000000	Padam5	Yes	78.43	Bacteria, Actinomycetota, Acidimicrobiia, Acidimicrobiales, SHLQ01, SHLQ01	4,866,565	61.58	1	98.29	2.99	37	4,614
					JAWPMT000000000	Padam50	No	75.58	Bacteria, Bacteroidota, Bacteroidia, Cytophagales, Cyclobacteriaceae, MS6	8,717,540	43.36	7	98.51	3.27	29	6,973
					JAWPMU000000000	Padam52	Yes	95.89	Bacteria, Pseudomonadota, Alphaproteobacteria, Rhodobacterales, Roseobacteraceae, Roseovarius, *Roseovarius pacificus*	3,452,676	62.46	1	95.84	0.1	4	3,049
					JAWPMV000000000	Padam53	Yes	75.32	Bacteria, Bacteroidota, Bacteroidia, J057, J057, JAGQVA01	9,489,248	42.27	5	99.45	2	62	7,493
					JAWPMW000000000	Padam54	Yes	76.36	Bacteria, Pseudomonadota, Gammaproteobacteria, UBA4575, UBA4575, UBA1858	2,402,513	38.95	1	95.32	1.27	552	2,421

^*a*^ –, not applicable.

## Data Availability

The raw reads have been submitted under the BioProject PRJNA996615 and the assemblies under accessions JAWPML000000000 (Padam16), JAWPMM000000000 (Padam2), JAWPMN000000000 (Padam27), JAWPMO000000000 (Padam3), JAWPMP000000000 (Padam36), JAWPMQ000000000 (Padam38), JAWPMR000000000 (Padam4), JAWPMS000000000 (Padam5), JAWPMT000000000 (Padam50), JAWPMU000000000 (Padam52), JAWPMV000000000 (Padam53), and JAWPMW000000000 (Padam54).

## References

[1] Oh ZG , Askey B , Gunn LH . 2023. Red rubiscos and opportunities for engineering green plants. J Exp Bot 74:520–542. doi:10.1093/jxb/erac349 36055563 PMC9833100

[2] Whitney SM , Baldet P , Hudson GS , Andrews TJ . 2001. Form I rubiscos from non-green algae are expressed abundantly but not assembled in tobacco chloroplasts. Plant J 26:535–547. doi:10.1046/j.1365-313x.2001.01056.x 11439139

[3] Gunn LH , Martin Avila E , Birch R , Whitney SM . 2020. The dependency of red rubisco on its cognate activase for enhancing plant photosynthesis and growth. Proc Natl Acad Sci U S A 117:25890–25896. doi:10.1073/pnas.2011641117 32989135 PMC7568259

[4] Zhou Y , Gunn LH , Birch R , Andersson I , Whitney SM . 2023. Grafting rhodobacter sphaeroides with red algae rubisco to accelerate catalysis and plant growth. Nat Plants 9:978–986. doi:10.1038/s41477-023-01436-7 37291398

[5] Lin MT , Hanson MR . 2018. Red algal rubisco fails to accumulate in transplastomic tobacco expressing Griffithsia monilis RbcL and RbcS genes. Plant Direct 2:e00045. doi:10.1002/pld3.45 31245711 PMC6508576

[6] Nelson JM , Hauser DA , Gudiño JA , Guadalupe YA , Meeks JC , Salazar Allen N , Villarreal JC , Li F-W . 2019. Complete genomes of symbiotic cyanobacteria clarify the evolution of vanadium-nitrogenase. Genome Biol Evol 11:1959–1964. doi:10.1093/gbe/evz137 PMC6645180 31243438

[7] Guppy. Oxford nanopore Technologies. Retrieved 4 Apr 2022. https://community.nanoporetech.com/

[8] Chen S , Zhou Y , Chen Y , Gu J . 2018. Fastp: an ultra-fast all-in-one FASTQ preprocessor. Bioinformatics 34:i884–i890. doi:10.1093/bioinformatics/bty560 30423086 PMC6129281

[9] Wick RR , Volkening J . 2018. Porechop: Adapter Trimmer for Oxford nanopore reads. doi:https://github.com/rrwick/Porechop

[10] Kolmogorov M , Yuan J , Lin Y , Pevzner PA . 2019. Assembly of long, error-prone reads using repeat graphs. Nat Biotechnol 37:540–546. doi:10.1038/s41587-019-0072-8 30936562

[11] Shen W , Le S , Li Y , Hu F , Zou Q . 2016. Seqkit: a cross-platform and ultrafast toolkit for FASTA/Q file manipulation. PLoS ONE 11:e0163962. doi:10.1371/journal.pone.0163962 27706213 PMC5051824

[12] Medaka: Sequence correction provided by ONT research.. PLOS Comput Biol. Accessed June 2023. doi:https://Github.com/Nanoporetech/Medaka

[13] Zimin AV , Salzberg SL . 2020. The genome polishing tool POLCA makes fast and accurate corrections in genome assemblies. PLOS Comput Bio 16. doi:10.1371/journal.pcbi.1007981 PMC7347232 32589667

[14] Wick RR , Holt KE . 2022. Polypolish: short-read polishing of long-read bacterial genome assemblies. PLOS Comput Biol 18:e1009802. doi:10.1371/journal.pcbi.1009802 35073327 PMC8812927

[15] Wood DE , Lu J , Langmead B . 2019. Improved metagenomic analysis with Kraken 2. Genome Biol. 20:257. doi:10.1186/s13059-019-1891-0 31779668 PMC6883579

[16] Kang DD , Li F , Kirton E , Thomas A , Egan R , An H , Wang Z . 2019. Metabat 2: an adaptive binning algorithm for robust and efficient genome reconstruction from metagenome assemblies. PeerJ 7:e7359. doi:10.7717/peerj.7359 31388474 PMC6662567

[17] Parks DH , Imelfort M , Skennerton CT , Hugenholtz P , Tyson GW . 2015. CheckM: assessing the quality of microbial genomes recovered from isolates, single cells, and metagenomes. Genome Res 25:1043–1055. doi:10.1101/gr.186072.114 25977477 PMC4484387

[18] Chaumeil P-A , Mussig AJ , Hugenholtz P , Parks DH , Hancock J . 2019. GTDB-Tk: a toolkit to classify genomes with the genome taxonomy database. Bioinformatics 36:1925–1927. doi:10.1093/bioinformatics/btz848 31730192 PMC7703759

[19] Langmead B , Salzberg SL . 2012. Fast gapped-read alignment with bowtie 2. Nat Methods 9:357–359. doi:10.1038/nmeth.1923 22388286 PMC3322381

[20] Tatusova T , DiCuccio M , Badretdin A , Chetvernin V , Nawrocki EP , Zaslavsky L , Lomsadze A , Pruitt KD , Borodovsky M , Ostell J . 2016. NCBI prokaryotic genome annotation pipeline. Nucleic Acids Res 44:6614–6624. doi:10.1093/nar/gkw569 27342282 PMC5001611

[21] Wick RR , Schultz MB , Zobel J , Holt KE . 2015. Bandage: interactive visualization of de novo genome assemblies. Bioinformatics 31:3350–3352. doi:10.1093/bioinformatics/btv383 26099265 PMC4595904

